# Suitable Camera and Rotation Navigation for People with Visual Impairment on Looking for Something Using Object Detection Technique

**DOI:** 10.1007/978-3-030-58796-3_57

**Published:** 2020-08-10

**Authors:** Masakazu Iwamura, Yoshihiko Inoue, Kazunori Minatani, Koichi Kise

**Affiliations:** 8 Institute Integriert Studieren, JKU Linz, Linz, Austria; 9 Jack Baskin School of Engineering, UC Santa Cruz, Santa Cruz, CA USA; 10 Dipartimento di Meccanica, Politecnico di Milano, Milan, Italy; 11 Support Centre for Students with Special Needs, Masaryk University Brno, Brno, Czech Republic; 12 Graduate School of Engineering, Osaka Prefecture University, Sakai, Japan; 13 National Entrance Examination Center, Tokyo, Japan

**Keywords:** Omnidirectional camera, Rotation navigation, Object detection.

## Abstract

For people with visual impairment, smartphone apps that use computer vision techniques to provide visual information have played important roles in supporting their daily lives. However, they can be used under a specific condition only. That is, only when the user knows *where the object of interest is*. In this paper, we first point out the fact mentioned above by categorizing the tasks that obtain visual information using computer vision techniques. Then, in *looking for something* as a representative task in a category, we argue suitable camera systems and rotation navigation methods. In the latter, we propose novel voice navigation methods. As a result of a user study comprised of seven people with visual impairment, we found that (1) a camera with a wide field of view such as an omnidirectional camera was preferred, and (2) users have different preferences in navigation methods.

## Introduction

For people with visual impairment, the lack of access to visual information can cause difficulty in their daily lives and decrease independence. To mitigate it, smartphone apps that can tell the user visual information have been developed. VizWiz [5] and Be My Eyes [4] are apps that enable people with visual impairment to ask remote sighted workers or volunteers in supporting them. EnVision AI [6], TapTapSee [12], interest by oneself. Let us confirm this. To take a photo of an object, the user has to know where it is. Of course, the purpose of using the apps is to know what it is. Hence, these apps are used only when “what (it is)” is unknown and “where (it is)” is known. Extending this idea, we find the following three types of visual information exist, as summarized in Table 1 and Seeing AI [11] are apps that use computer vision techniques [8] to obtain visual information. As of the time of writing this paper, many people with visual impairment use these apps except VizWiz.

**Table 1.** Categorization of visual information obtained by computer vision techniques. “What” and “where” indicate *“what it is”* and *“where it is,”* respectively.

	Condition	Representative task	Required tools and techniques
(i)	“What” is unknown. “Where” is known.	Obtaining the visual information on the object that the user photographs	Current smartphone apps that use computer vision techniques such as [6, 11, 12] can be used.
(ii)	“What” is known. “Where” is unknown.	Looking for something	It is better to use a camera with a wide FoV such as a fisheye camera and an omnidirectional camera.
(iii)	“What” is unknown. “Where” is unknown.	Finding something valuable and unexpected to the user	It is better to use a camera with a wide FoV, and the information provided to the user should be selected.

This paper focuses on the latter approach, i.e., the apps that use computer vision techniques. While it has not been argued before, they can be used under a specific condition only. It is only when *the user can photograph the object of interest by oneself*. Let us confirm this. To take a photo of an object, the user has to know *where it is*. Of course, the purpose of using the apps is to know *what it is*. Hence, these apps are used only when *“what (it is)”* is unknown and *“where (it is)”* is known. Extending this idea, we find the following three types of visual information exist, as summarized in Table 1.

- **Category (i)—** ***what is unknown and where is known.*** 
  In this category, the user can photograph the object of interest by oneself. This type of visual information can be obtained by the current smartphone apps that use computer vision techniques such as [6, 11, 12].
- **Category (ii)—** ***what is known and where is unknown.*** 
  A representative task of this category is *looking for something.* That is, the user knows *what the user is looking for*, but does not know *where it is*. As the user does not know where the object of interest is, the user cannot use the current smartphone apps in the same way as category (i). It is because the user needs to move the smartphone here and there to take a photo of the object. Hence, it is expected that using a camera with a wide field of view (FoV), such as a fisheye camera and an omnidirectional camera, is better. As the user already knows *what it is*, differently from category (i), the app is expected to tell only *where it is* if found.
- **Category (iii)—** ***both what and where are unknown.*** 
  In this category, the user does not expect that the app will provide any visual information to the user. However, if provided, the information is expected to be valuable to the user. Concept-wise, it is similar to the recommendation system used in e-commerce websites such as Amazon.com, because it is expected to introduce products that are potentially interesting and unexpected to the user. Thus, a representative task is finding something valuable and unexpected to the user. In the real world scenario, the app is required to obtain as much visual information all around the user as possible. Hence, similar to category (ii), it is expected that using a camera with a wide FoV is better. A big difference from other categories is that the amount of visual information potentially provided by the app can be much. In other words, the app may find multiple objects valuable to the user simultaneously. However, too much information is just annoying. Hence, the amount of visual information to be provided to the user must be controlled.

Among them, we focus on category (ii) and argue *looking for something*, which is a representative task of the category, in the following two issues.

The first issue is about cameras. In the task, we assume the user looks for a designated object around the user using an app that uses a computer vision technique to detect the object and guides the user to reach the target object. As the system needs to capture the object with the camera, the task is expected to become easier by using a camera with a wide FoV, such as a fisheye camera and an omnidirectional camera. Hence, in a user study, we investigate if our expectation regarding the cameras is correct.

The second issue is about rotation navigation methods. In turn-by-turn navigation, Ahmetovic et al. [3] have studied rotation errors and found that the participants tend to over-rotate the turns, on average, 17$^{\circ }$ (hereafter, deg.) more than instructed. They have concluded that simply notifying the user when the user reaches the target orientation, like they did in the research, is error prone, and a different interaction, such as continuous feedback, is required. As a follow up, Ahmetovic et al. [1] have investigated three sonification techniques to provide continuous guidance during rotation. However, it is not necessary to instruct by sound. Hence, we introduce three voice instructions and investigate the users’ preferences in the user study.

## Method

### Prototype System

In *looking for something*, we implement a computer-vision-based prototype system that guides the user to reach the target object in a step-by-step manner.

- **Step 1: Object detection**  The system detects an object of the designated category in the captured image. In the user study, we designated easy-to-detect object categories, but only one instance existed in the room, such as a *laptop* and a *bottle*. Once the object detection method outputs the bounding box of the target object, the direction of the target object from the user is recorded.
- **Step 2: Rotation navigation**  The user rotates on the spot until the target object comes in front. By comparing the output direction of the electronic compass with the direction of the target object, the system guides the user to rotate using a rotation navigation method.
- **Step 3: Forward navigation**  With the guidance of the system, the user advances toward the target object and stops in front of the object. It uses the depth camera to measure the distance to the target object, and speaks the distance periodically, like “1.5 m, 1.3 m, ...” It ends when the user reaches a distance of 0.8 m.

**Fig. 1.**
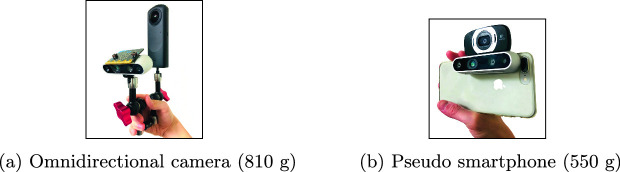
Camera systems used in the user study. They were comprised of a depth camera and an electronic compass, in addition to (a) an omnidirectional camera or (b) a web camera attached to a smartphone.

The implemented prototype system consisted of a laptop computer (MacBook Pro) and a camera system in Fig. 1. As shown in Fig. 1(a), one consisted of an omnidirectional camera (Ricoh Theta Z1) used in Step 1 of the above procedure, an electronic compass (Freescale MAG3110 installed on BBC micro:bit), and a depth camera (Intel RealSense D435). The electronic compass was used in Step 2 to quickly sense the user’s direction and promptly give the user feedback. The depth camera was used in Step 3 to measure the distance to the target object. The other was a pseudo smartphone shown in Fig. 1(b). Instead of the smartphone’s embedded camera, we used a web camera (Logicool HD Webcam C615) in Step 1. We used the same electronic compass and depth camera for a fair comparison. To detect the target object, we ran a PyTorch implementation [13] of *you only look once* (YOLO) version 3 [10], which is a representative object detection method, on the laptop computer. It was trained on COCO dataset [9] consisting of 80 object categories. As the object detection method assumes to input a perspective image, the image captured with the omnidirectional camera was converted to eight perspective images in the same manner as [7]. The prototype system speaks its current state, like “Searching an object. Please stay and wait.” “Detected.” “Measuring the distance.” and “The object exists near you.”

### Existing Rotation Navigation

Ahmetovic et al. [1] have introduced the following three sonification techniques that provide continuous guidance during rotation.

- **Intermittent sound (IS)** triggers impulsive “beeping” sounds at a variable rate, which is inversely proportional to the angular distance, like a Geiger-Müller counter.
- **Amplitude modulation (AM)** employs a sinusoidal sound, modulated in amplitude by a low frequency (sub-audio) sinusoidal signal. The frequency of the modulating signal is inversely proportional to the angular distance, producing a slowly pulsing sound at large angular distances, which becomes stationary when the target is reached.
- **Musical scale (MS)** plays eight ascending notes at fixed angular distances while approaching the target angle.

They concluded that IS and MS when combined with Ping (impulsive sound feedback emitted when the target angle is reached) were the best with regard to rotation error and rotation time.

### Proposed Rotation Navigation

We examine the following five (three voice and two sound) navigation methods.

- **Left or Right (LR)** repeatedly (approximately 1.5 times per second) tells the direction toward the target object, i.e., “Left” or “Right.” When the target object comes within 15$^{\circ }$. in front of the user, it tells “In front of you.”
- **Angle (AG)** repeatedly tells the relative rotation angle to the target object, followed by “Left” or “Right.” The front of the user is always regarded as 0$^{\circ }$. For example, if the target object exists at an angular distance of 60$^{\circ }$. On the right-hand side of the user, the system speaks “60$^{\circ }$, right.” After the user rotates by 15$^{\circ }$, it speaks “45$^{\circ }$, right.” In front of the target object (within 15$^{\circ }$), it tells “In front of you.”
- **Clock Position (CP)** is similar to AG but uses the clock position. Taking the same example as AG, it speaks “2 o’clock.” In front of the target object (within 15$^{\circ }$), it tells “In front of you.”
- **Intermittent Beep (IB)** is similar to IS of [1]. It triggers impulsive “beeping” sounds at a variable rate, which is inversely proportional to the angular distance. The rates in the front (15$^{\circ }$) and back (180$^{\circ }$) were approximately 5 Hz and 1.2 Hz, respectively. IB is designed to use earphones; beeps are played on only the left or right earphone to indicate the rotation direction. When the target object comes within 15$^{\circ }$ in front of the user, it plays beeps sounds at a rate of approximately 8 Hz on both earphones.
- **Pitch (PT)** plays sounds with a variable pitch. In our implementation, the front and back pitches were 1570 Hz and 785 Hz (six and three times of C4 in scientific pitch notation), respectively. In contrast with MS of [1] that plays eight discrete notes, PT plays continuous notes. Same as IB, PT plays sounds on only the left or right earphone to indicate the rotation direction. In front of the target object, PT behaves in the same manner as IB.

## User Study

We performed a user study comprised of seven people with visual impairment. As summarized in Table 2, the participants consisted of four males and three females, ages 23 to 48. Six were totally blind, and one had low vision. The user study consisted of the following four parts.

**Table 2.** Participants’ demographic information. “OA” denotes “Onset age.”

ID	Age	Sex	Visual impairment	OA
A	23	F	Totally blind	2
B	27	M	Totally blind	5
C	27	F	Totally blind	7
D	48	M	Low vision (left: blind, right: 0.03, narrowing of visual field)	10
E	48	M	Totally blind	6
F	27	M	Totally blind	10
G	34	F	Totally blind	13

**Table 3.** Questions and answers of pre-study interview.

Question	A	B	C	D	E	F	G
Q1. Do you live together with someone?	Yes	No	Yes	No	Yes	Yes	Yes
Q2. If yes in Q1, is the person(s) sighted?	Yes	–	No	–	Yes	No	No
Q3. How often do you look for something?	1	1	1	1	1	1	1
(1: Every day, 2: 3–4 days per week, 3: once a week, 4: once a month, 5: several times per year)							
Q4. How often do you encounter trouble in looking for something?	2	3	4	3	2	2	5
(1: Every day, 2: 3–4 days per week, 3: once a week, 4: once a month, 5: several times per year)							
Q5. Where do you mostly look for something? ([multiple choice] 1: home, 2: office or school, 3: store, 4: other)	1,2	1	1	1,4 (outside)	1,4 (outside)	1	1
Q6. How do you look for something? (1: grope, 2: ask a sighted person, 3: use a smartphone app, 4: other)	1,2	1,2	1	1,2	1,2	1	1
Q7. What item do you mostly look for?	See Table 4						
Q8. How long does it take to find lost stuff?	3	1,2	1	3	3	2	2
(1: within 1 min., 2: 1–5 min., 3: more than 5 min.)							
Q9. Where do you find lost stuff?	See Table 5						
Q10. What causes you to look for something?	2	1,2	2	1,2	1,3	1	4
(1: wrongly remember where the lost stuff is placed, 2: do not remember where the lost stuff is placed, 3: the stuff is moved without knowing it, 4: other)							
Q11. Do you have any idea of how to avoid looking for something? ([multiple choice] 1: keep the room clean, 2: fix the place, 3: use an IC tag, 4: other)	2	2	2	2	2	1,2	1,2

**Table 4.** [Pre-study Interview] Answers to Q7 “What do you mostly look for?”

ID	Answers
A	Smartphone, and braille notetaker. I always give up looking for hairpins and hair rubber bands.
B	Smartphone, earphones, charger, and credit card.
C	Smartphone.
D	Smartphone for work.
E	Something dropped. Remote controller for TV.
F	Prepaid transportation card, and earphones.
G	Smartphone, keys, and slipper.

**Table 5.** [Pre-study Interview] Answers to Q9 “Where do you find lost stuff?”

ID	Answers
A	On a chair or table. Sometimes on the floor. Lost stuff is merely covered by something.
B	In the pocket of a jacket which is not usually worn. In a bag pocket.
C	In many cases, within arm’s reach. I often encounter difficulty in finding stuff that is neatly placed on the table.
D	In the pocket of a jacket or bag.
E	As the person living together moves stuff, I find where it is moved.
F	On the table or in a bag. Otherwise, in the pocket of a jacket.
G	In many cases, on the floor.

***1. Instruction of the experiments*** 

We told the participants that our research topic was *looking for something* and gave a brief overview of the experiments.

***2. Pre-study interview: Survey on looking for something*** 

We asked the participants about looking for something. This interview was performed for every two persons except A. That is, the interview groups were [A], [B and C], [D and E], and [F and G]. The questions and answers are summarized in Table 3. Answers of Q7 and Q9 are shown in Tables 4 and 5.

The answers of the participants are summarized as follows. Five out of seven participants lived together with someone (Q1). Among them, two lived with sighted persons (Q2). While they all looked for something every day (Q3), they did not encounter trouble every day (Q4). They all looked for something at home, and three did it in other places (office or school, and outside) (Q5). They all groped to look for something, expecting to find it in arm’s reach, while four asked a sighted person if available (Q6). Five mostly looked for a smartphone, while earphones and other stuff were also often looked for (Q7). Required time to look for lost stuff was of variety (Q8). Some answered that they gave up looking for if it took more than 5 min. (Q8). The lost stuff was found in the pocket of a jacket and a bag, and on a chair and a table (Q9). Losing stuff was mostly caused by wrongly remembering and forgetting where it was placed (Q10). They all answered that their remedy to avoid losing stuff was to fix the place, while two answered to keep the room clean (Q11).

**Fig. 2.**
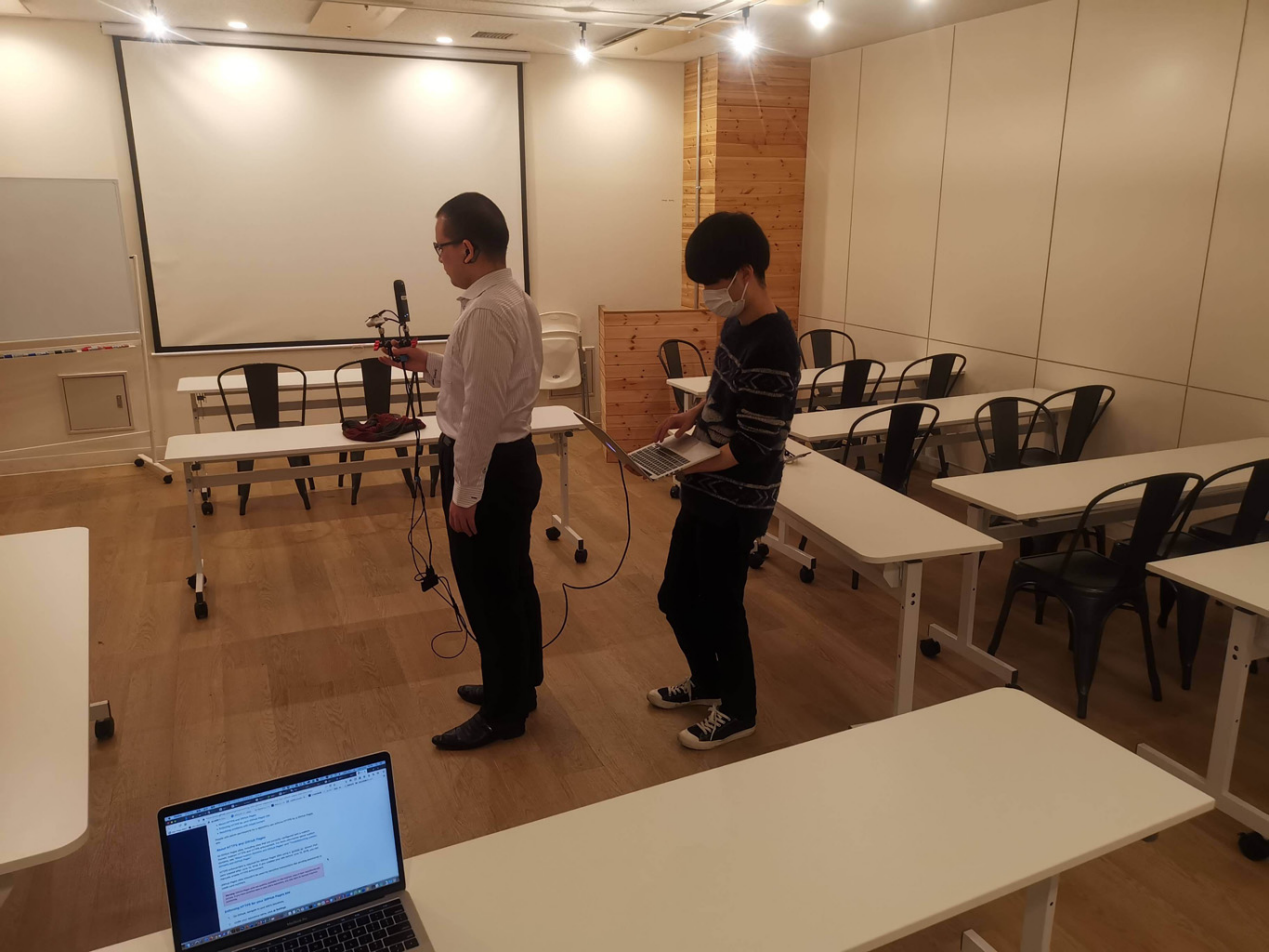
A snapshot during the experiment. The experimenter holding the laptop computer stands behind the participant to prevent the camera from capturing him. In this case, the laptop computer at the bottom was the target object.

**Table 6.**
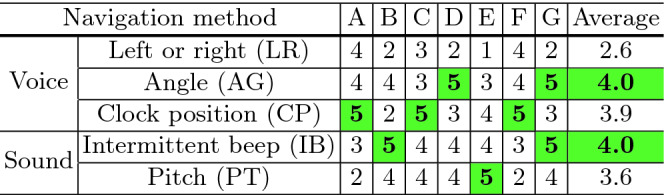
Evaluation of navigation methods on a 5-point scale.

***3. Experiment 1: Comparison of five rotation navigation methods*** 

Differently from the pre-study interview, the following two experiments were performed for each participant. In this experiment, we asked participants to use five rotation navigation methods one by one through Steps 1 (object detection using the omnidirectional camera) and 2 (rotation navigation) in Sect. 2.1. As IB and PT were designed to use earphones, for a fair comparison, participants used earphones for all navigation methods. Figure 2 shows how the experiment was performed. Table 6 shows their preferences on a 5-point scale, in which a large number means better. Besides, their comments on the five navigation methods and ideas about easy-to-use navigation methods are shown in Tables 7, 8, 9, 10, 11 and 12.

**Table 7.** [Experiment 1] Feedback on Left or Right (LR).

ID	Answers
A	Though I could not see how much I should rotate, I could try to rotate much.
B	Simple but not intuitive. As I could not see how much I should rotate, I over-rotated.
C	I could get the rotation direction, but could not see how much I should rotate.
D	The most primitive.
E	I felt anxious, as I could not see how much I should rotate. I needed to concentrate on listening.
F	Simple and intuitive. Though I could not see how much I should rotate, this way is easy to use.
G	I thought this way was simple and easy to get in the instruction. However, as I could not see how much I should rotate, I felt it was unreliable. I think it can get better with improvement.

**Table 8.** [Experiment 1] Feedback on Angle (AG).

ID	Answers
A	The resolution in angle was too detailed. I prefer CP.
B	While the resolution was too detailed, this way was easy to get, as the angle is absolute.
C	Though it seemed not to cause trouble and easy to get, it was not easy for me to imagine how much I should rotate.
D	If I get used to this way, it would be the safest choice.
E	I needed to be strategic. It took some time to think about how much I should rotate after hearing the angle.
F	Simple. Though I could get the angle, I could not immediately imagine how much I should rotate. I may need to get used to it.
G	It was easy to get when I needed to stop, as spoken angles were decreasing.

**Table 9.** [Experiment 1] Feedback on Clock Position (CP).

ID	Answers
A	This way was the easiest to get.
B	Once it says “5 o’clock,” it should keep saying “5 o’clock” even if I rotate. Or, it is easier for me to get if (by fixing the target at the 12 o’clock position) it says “you are now at the 5 o’clock position.” Maybe I need to get used to it.
C	This way was easy to imagine both rotation direction and angle.
D	I need to get used to this way.
E	As I am used to this way, I could imagine how much I should rotate. But, the resolution in angle maybe too rough.
F	As I am used to the clock position, this way was very easy to understand, so that I could reach the target direction immediately.
G	I needed to think about which direction 5 o’clock is. It is because I am not used to it.

**Table 10.** [Experiment 1] Feedback on Intermittent beep (IB).

ID	Answers
A	Different from what I imagined. Using a 3D audio effect, hearing the sound from the target direction is more intuitive. I felt the target always existed on the side.
B	Intuitively, this way was the easiest for me. It was easy to rotate toward the direction I could hear the sound. This way is close to an audio game for the blind. Using both ears is negative.
C	Intuitively, it was easier to get, compared to LR.
D	Everyone would be able to get this way.
E	Though this way was intuitive, it took time to sense the time difference between two beeps, which would negatively affect when I rotate fast.
F	As hearing the sound in either of my ears made me confused, voice navigation was better. Though I could not get how much I should rotate, I could notice that I rotate too much when I heard the sound in the other ear.
G	I had the impression that I was approaching the target. I felt it was trustable.

**Table 11.** [Experiment 1] Feedback on Pitch (PT).

ID	Answers
A	The pitch of the first sound was too low. To me, the high pitch did not link to getting close to the target.
B	The pitch of the last sound was too high. Compared to IB, it was not easier to expect the target angle. Using both ears is negative.
C	Intuitively, it was easy to get, even without hearing a voice. It would be usable in a noisy place.
D	I like this way, while I think this way requires a sense of pitch. This way is used in a screen reader (NVDA).
E	This way was the best among the five methods. I could get feedback immediately. I could find the target sensuously.
F	As hearing the sound in either of my ears made me confused, voice navigation was better. I could not imagine how much I should rotate, as I could not see how the pitch became when I approached the target. I could notice that I rotated too much when I heard the sound in the other ear.
G	Though this way was easy to get, I could not distinguish sounds in detail. If it takes long to find the target object, my ears will hurt.

**Table 12.** [Experiment 1] Idea about easy-to-use navigation methods.

ID	Answers
A, D	I have no idea.
B	Use of sound volume, 3D audio effect, and the interval of vibration.
C	The vibration of the camera, which makes earphones unnecessary, while telling left or right maybe not easy.
E	To be strategic, it is better to tell the angle first. Then, using a sensuous method such as the duration or interval of vibration. It is also ok that a band wrapped around the belly tells the direction by vibrating the target direction part.
F	While vibration is a possible solution, I think CP is the best.
G	Voice with vibration would be able to be used in a noisy place.

**Table 13.**
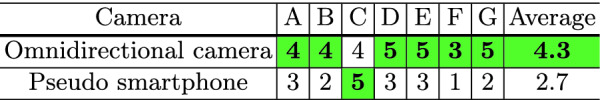
Evaluation of a camera on a 5-point scale.

Table 6 shows that the participants’ preferences were of variety. That is, all navigation methods except LR were selected as the best by at least one participant. Related results are reported in two papers; musical experience affects the users’ behavior [1]; expertise affects interaction preferences in navigation assistance [2]. In our experiment, while we did not ask their expertise, from their comments^1^, we can see that the participants have their compatibility with navigation methods. These imply that no single best method for everyone exists, and personalization of user interfaces is vital. We also asked the participants if they hesitate to wear earphones on both ears, and found that one (D) did not hesitate, four (A, C, F, and G) did not if they are at home, two (B and E) did.

**Table 14.** [Experiment 2] Comments on the omnidirectional camera.

ID	Answers
A	It was a bit heavy. If it was not heavy, it was the most convenient.
B	To find the object, I did not have to rotate. Even so, I would buy the smartphone.
C	While it seems convenient and I think it can find the stuff quicker, requiring a particular device (i.e., omnidirectional) and its heaviness were negative points.
D	It was unexpectedly good, as it told me how far in the angular distance to the object, and found the object earlier than the smartphone.
E	It was good, as it told me the direction of the target object.
F	It was heavy. Finding the bottle took time more than I expected. While it found the laptop computer quickly, the “front” the system said was slightly different from my real front.
G	It was convenient, as I did not have to rotate. It was more accurate and quicker than I expected. I want to use this. The distance to the object was not important.

**Table 15.** [Experiment 2] Comments on the pseudo smartphone.

ID	Answers
A	If I have to find the object by moving the smartphone, I prefer to grope. The response was slow. Quicker is better.
B	I had to rotate to find the object. Even if the system did not find the object, I could not judge if it exists in the room (the omnidirectional camera is the same).
C	An advantage is easy to introduce, as I can use my smartphone. Easy to hold.
D	I expected the smartphone was better. However, I needed to adjust the angle.
E	The system could not find the object unless it captures it, which frustrated me. As I could not see how quickly the system processed an image, I could not see how fast I could rotate. While it was faster than groping, it took time.
F	While the camera was not heavy, it is not suitable for looking for something. In real use, if I can roughly guess the direction of the object, I may be able to use this. If not, groping is better.
G	It was hard to capture the target object, as I needed to take care of horizontal rotation and vertical rotation. I prefer to grope.

***4. Experiment 2: Selection of camera*** 

We asked participants to use each of the two camera systems and complete the 3-step finding process in Sect. 2.1. They used the best navigation method selected in experiment 1 for each participant but had the freedom to use or not to use earphones. Table 13 shows an omnidirectional camera was preferred by six, while the pseudo smartphone by one. Tables 14 and 15 show the participants’ comments on the camera systems. Six (all but C) commented on the difficulty of using the pseudo smartphone in *looking for something*. In contrast, they all, including participant C who preferred the pseudo smartphone, found advantages of the omnidirectional camera, while three (A, C, and F) commented its heaviness. Hence, we conclude the omnidirectional camera has advantages in the task.

## Conclusions

In this paper, we focused on apps that use computer vision techniques to provide visual information. We pointed out that the current smartphone apps can only be used under a specific condition, and categorized the tasks of obtaining visual information into three. As a representative task of a category, we focused on *looking for something*. In the task, we proposed a prototype system that used an omnidirectional camera and the use of voice in rotation navigation. A user study comprised of seven people with visual impairment confirmed that (1) a camera with a wide FoV is better in such a task, and (2) users have different preferences in rotation navigation. The latter implies that no single best method for everyone exists, and it is vital to personalize user interfaces.

## Footnotes

1 Let us highlight some comments. **Participant B on CP:** He preferred to put the target, instead of himself, at the 12 o’clock position. **Participants C, E, and G:** C and E were not good at AG but good at CP, while G was the opposite. **Participant A on IB:** As he imagined a 3D audio effect, hearing the sound played on only left or right, he felt the target object was on the side.
